# Analysis of Pickled Cucumber Products, Based on Microbial Diversity and Flavor Substance Detection

**DOI:** 10.3390/foods13081275

**Published:** 2024-04-21

**Authors:** Xiaoyue Tang, Xiangyu Chen, Fuxiang Li, Mengmeng Huang, Lele Xie, Jingping Ge, Hongzhi Ling, Keke Cheng

**Affiliations:** 1 Engineering Research Center of Agricultural Microbiology Technology, Ministry of Education & Heilongjiang Provincial Key Laboratory of Plant Genetic Engineering and Biological Fermentation Engineering for Cold Region & Key Laboratory of Microbiology, College of Heilongjiang Province & School of Life Sciences, Heilongjiang University, Harbin 150080, China; m15045092750@163.com (X.T.); hmm1298372263@126.com (M.H.); 2221601@s.hlju.edu.cn (L.X.); gejingping@126.com (J.G.); 2 Engineering Research Center of Health Food Design & Nutrition Regulation, School of Chemical and Engineering and Energy Technology, Dongguan University of Technology, Dongguan 523808, China; rainchen03@163.com (X.C.); fuxianglee93@163.com (F.L.)

**Keywords:** fermentation, Gas Chromatography and Ion Mobility Spectrometry (GC–IMS), microbial community, volatile

## Abstract

Changes to the microbial community during pickled cucumber fermentation were studied using the 16S rDNA technique. The changes of volatile organic compounds (VOCs) during pickled cucumber fermentation were studied by gas chromatograph–ion mobility spectrometry. At the phylum level, *Cyanophyta* and *Proteobacteria* were the dominant flora in the natural fermentation group, and *Firmicutes* were the dominant flora in the added-bacteria fermentation group. At the generic level, the addition of *Lactobacillus* led to changes in the community of the bacteria in the added-bacterial fermentation group and decreased the species abundance of other bacteria. In total, 75 volatile organic compounds were identified from naturally fermented pickled cucumber, and 60 volatile organic compounds were identified from fermented pickled cucumber with bacterial addition. The main metabolites were esters, aldehydes, acids, alcohols, ketones, alkanes, nitriles, and alkenes. These metabolites will bring their unique aroma components to the pickled cucumber. Metabolomic analysis of the O2PLS model showed that *Weissella* and *Lactobacillus* were closely and positively correlated with nine alcohols, six esters, five aldehydes, four acids, three ketones, and one pyrazine. *Pseudomonas* and *norank_f_Mitochondria* show a close positive correlation with four kinds of alcohols, two kinds of esters, one kind of aldehyde, and one kind of nitrile.

## 1. Introduction

Pickled cucumber, a kind of pickle that tastes sour and sweet, is a Chinese traditional appetizer. Residents will harvest cucumbers with salt water, made into pickled cucumbers, in the winter to eat. Pickled cucumber has the functions of beauty, weight loss, and blood lipid lowering. Pickled cucumbers have a long history in different countries; they have long been one of the most relevant primary retail fermented vegetable products produced in the United States and Europe [1]. Pickled cucumbers are mainly fermented spontaneously by adding spices, salt, and water to the cucumbers at a certain temperature. The process of making pickled cucumbers in China is still based on natural fermentation, which provides the right conditions for detrimental microbial growth [2]. These micro-organisms will produce harmful metabolites and toxins during fermentation, which will cause serious harm to the quality of the final product and the health of consumers [3]. With the continuous improvement of people’s living standards, the flavor of pickled cucumber has received increasing attention.

A few researchers have studied the volatile compounds in cucumbers. Zhang et al. [4] used HS-SPME/gas chromatograph–mass spectrometry (GC–MS) technology to detect volatile compounds in fresh cucumbers, and the results revealed that (E, Z)-2,6-nonadienal, (E)-2-nonenal, 2,4-heptadienal, lauraldehyde, hexanal, and caryophyllene, among other aroma compounds, were the main volatile components in cucumber. A total of 314 components were identified, and 137 volatile compounds in fermented cucumber brine were identified [5]. Marsili et al. [6] found that trans-4-hexenoic acid and cis-4-hexenoic acid are the main odor-influencing substances in fermented cucumber brine. Palma-Harris et al. [7] found that the most relevant chemicals affecting cucumber flavor are (E, Z)-2,6-nonadienal and 2-nonenal (E, Z). However, these conclusions are mainly based on the studies of some researchers and the lack of a certain representativeness. GC–IMS is highly sensitive and can be used to characterize volatile compounds due to its short analysis time, rapid analysis, wide use, and accurate separation of volatile compounds from complex samples [8]. Currently, among the instruments commonly used in the detection of volatile components of food, there are advantages and disadvantages in the detection speed, sensitivity, and convenience, and GC–IMS is another technology in the field of food flavor analysis and quality detection [9].

Advances in next-generation sequencing technology have enabled high-throughput DNA sequencing to help us better research the microbial diversity in the object under test. This technology, provided to users by the Illumina platform, is used to research the microbiome in food [10]. Pickled cucumbers are fermented in closed jars, and the traditional pickled cucumber fermentation process is dominated by *Lactobacilli*. *Lactobacillus pentosus*, *Lactobacillus plantarum*, *Weissella* spp., *Leuconostoc* spp., and *Lactococcus* spp. were also found in pickled cucumbers [11]. Pérez-Díaz et al. [12] found some insignificant microbiomes in the brine of pickled cucumbers, such as *Pseudomonas*, *Pantoea*, *Stenotrophomonas*, and *Acinetobacter*. In the process of pickled cucumber fermentation, the catabolism of micro-organisms in the formation of volatile metabolites plays a vital role. However, regarding the fermentation process of pickled cucumber, fragrance ingredients, and flavor difference change, research is limited, and there are no specific studies on the transformation of micro-organisms during pickled cucumber fermentation. Thus, the underlying mechanisms of microbial formation and flavor VOCs in pickled cucumbers are still poorly known.

The main purpose of this study was to detect and analyze the microbial diversity and volatile compounds during the fermentation of pickled cucumber to better explain the dynamic change of the bacterial community and the formation mechanism of VOCs in pickled cucumber. The Operational Taxonomic Unit (OTU) clustering method was used to analyze the microbial diversity of pickles fermented by natural fermentation and added bacteria, and the dominant bacterial groups in pickled cucumber were determined by Linear discriminant analysis Effect Size (LEfSe) analysis. In the present study, VOCs produced by natural fermentation and added-bacteria fermentation pickled cucumber samples were detected by GC–IMS, and the characteristic fingerprints were established. This study provides a theoretical basis for future studies on the metabolites and flora structure of pickled cucumber.

## 2. Materials and Methods

### 2.1. Samples

Cucumbers were purchased from the local central red moon supermarket in Harbin, Heilongjiang Province, and the variety was Nasha. *Lacticaseibacillus paracasei* HD1.7 was added for fermentation. This strain was separated from Northeast Sauerkraut in our laboratory. MRS medium was used to activate *Lacticaseibacillus paracasei* HD1.7 and cultured at 37 °C for 24 h. Pickled cucumber was achieved by natural fermentation and added-bacteria fermentation. The natural fermentation group was classified as Z, and the added-bacteria fermentation group was classified as J. First, the dirt on the surface of the cucumber was washed with distilled water, and the root was sorted out and cut into 8 cm × 1 cm × 1 cm (length × width × thickness) with a regular shape and consistent size. The weight of cucumbers in each can was 800 g. In total, 4% salt, 4% sugar, and 1% spices (garlic, chili, and pepper) were added to the cucumbers and placed into a sealed tank of 10 cm × 10 cm × 10 cm for sealed fermentation at 18℃. The added-bacteria fermentation was the same as that before natural fermentation, *Lacticaseibacillus paracasei* HD1.7 was added at a 1% inoculated amount before sealed fermentation, and the cell density was 10^7^ CFU/mL. The parallel test conditions were the same as above, i.e., three groups of parallel samples were pickled at the same time and in the same environment.

### 2.2. Detection and Analysis of Volatile Compounds

Using GC–IMS (FavorSpec^®^ Static Headspace Gas Chromatography and Ion Mobility Spectrometry), the Analysis Sensor System MBH (G.A.S.) instrument of the Dortmund Association, Germany, Purchased from Hanon Advanced Technology Group Co., Ltd.), the pickled cucumber was analyzed using a gas chromatograph–ion mobility spectrometer (FavorSpec, G.A.S. Instrument, Dortmund, Germany) equipped with an FS-SE-54-CB-1 capillary column (15 m × 0.53 mm × 1 μm), modified with reference to the method of Liu et al. [13].

An amount of 2 mL of the sample was put into a 20 mL headspace glass sample bottle and incubated at 60 °C and 500 rpm for 15 min. Subsequently, the 85 μL headspace was automatically injected with a heated syringe at 60 °C. The column was maintained at 40 °C, and the drift tube temperature was maintained at 150 °C. High-purity nitrogen was selected to act as the sample gas. Each spectrum was recorded as the average of 12 scans. To avoid cross-contamination, the syringe is automatically flushed with nitrogen for 30 s to 5 min before and after each analysis. All tests were repeated three times. The retention index (RI) was calculated with n-ketone C4-C9 (Sinophosphoric Chemical Reagent Beijing Co., Ltd., Beijing, China) as the standard. The LAV (version 2.2.1) analysis software of GC-IMS was used to collect the volatile flavor components of pickled cucumber, establish the fingerprint, and analyze the characteristic flavor components. The VOCs collected were qualitatively analyzed by the NIST14 vapor retention index database and IMS migration time database built into GC × IMS Library Search qualitative software (version 1.0.3).

### 2.3. Bioinformatics Methods and Microbial Diversity Analysis

Species difference and network function prediction analyses were performed on the bacterial community data to explore the correlation between species genes and metabolism. PICRUSt software was used to analyze the functional composition of sample bacterial communities in amplification sequencing results during fermentation.

### 2.4. Analysis of Network Construction

The relationship between micro-organisms in naturally fermented pickled cucumber samples was studied using a single-factor correlation network diagram. The network map revealed significant relevance between different genera of the bacterial community in the sample. Nodes in the network diagram represent genera, and edges correspond to correlations between nodes. The key genus is the genus that has the highest central value in the network [14].

### 2.5. Analysis of Microbial Function

The OTU abundance table was standardized by Phylogenetic Investigation of Communities by Reconstruction of Unobserved States (PICRUSt), and OTU information was obtained from the corresponding green gene ID of each OTU. Specific information on bacterial metabolic pathways was consulted in KEGG’s database, and the abundance of each functional class was calculated based on OTU abundance and analyzed.

### 2.6. Data Analysis

An O2PLS model was established using SIMCA-14.1 to forecast potential correlations between core bacterial communities and flavor-volatile components. There are many models used to analyze metabolomics, such as PCA, OPLS, OPLS-DA, and O2PLS. O2PLS is generally used to analyze the degree of difference between two different groups. SPSS (version 25.0), Cytoscape, AMOS (version 23.0), R (version 3.6.3), and other software, as well as the Majorbio platform, were used to process the data. The experimental data were denoted as X ± SD, and *p* < 0.05 was considered a significant difference.

## 3. Results and Discussion

### 3.1. Changes in Bacterial Community Structure in Pickled Cucumber Samples

**Figure 1:**
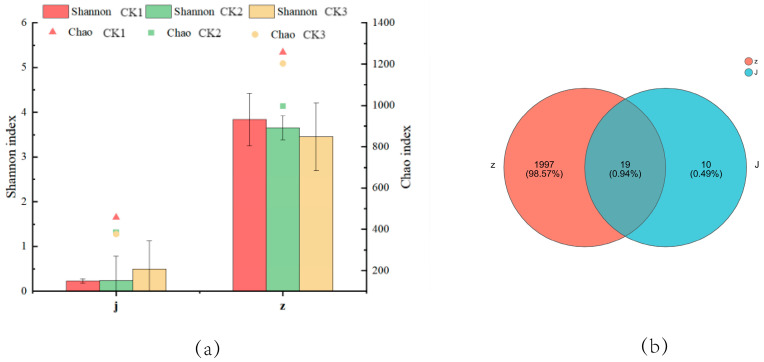
Changes in bacterial community structure in pickled cucumber. (**a**) Shannon and Chao indices of J&Z for samples. CK1, CK2, and CK3 were parallel samples between control groups. (**b**) OTU number distribution of J&Z samples.

The coverage index of samples is > 0.99 (Figure 1a), suggesting a high detection rate of species in the samples, which can reflect the real situation of micro-organisms in the samples. The Chao and Shannon indices of the natural group are significantly higher than those of the added-bacteria group in terms of overall trend, suggesting that the α diversity of the natural group is richer than that of the added-bacteria group. Studies have shown that Lactobacillus is a safe natural preservative and can produce a variety of antimicrobial compounds [15]. Therefore, the α diversity of the added-bacteria group is low because the growth and reproduction of Lacticaseibacillus paracasei HD1.7 after exogenous addition will significantly inhibit other populations, which will reduce the bacterial community richness in pickled cucumber.

The OTU data of the obtained samples were analyzed by a Venn diagram (Figure 1b). In the five fermentation stages, the number of OTU unique to the natural group was 1997; it accounts for 98.57% of the total OTU; the number of OTU unique to the added-bacteria group was 10, accounting for 0.49% of the total number of OTU; and the number of OTU shared by the natural and added-bacteria groups was 19. Accounting for 0.94% of the total number of OTUs, this is consistent with the Chao and Shannon index results we mentioned above. This indicates that the addition of Lacticaseibacillus paracasei HD1.7 can indeed change the number of bacterial communities in the fermentation system and reduce the diversity of bacterial communities during pickled cucumber fermentation.

#### Bacterial Composition of Pickled Cucumber

**Figure 2:**
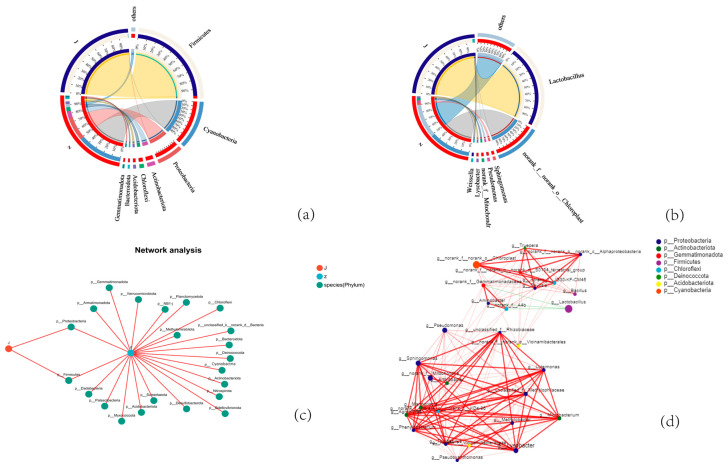
Microbial diversity analysis of pickled cucumbers. (**a**) Phylum-level bacterial community composition during fermentation. (**b**) Genus’s level bacterial community composition during fermentation. (**c**) Collinear network of phylum level during fermentation. (**d**) Species univariate correlation network Z: natural fermentation group; J: added-bacteria fermentation group.

The changes in the composition of the bacterial community at the gate level on the third day of natural and added-bacteria fermentation pickled cucumber are shown in Figure 2a. Firmicutes (e.g., Lactobacillaceae, Enterobacteriaceae, Bacillus, and Clostridium) in the added-bacteria fermentation group were identified as the dominant phyla. According to the quantitative detection, Firmicutes accounted for >97% of all phyla abundance in the added-bacteria fermentation group, and the phyla abundance composition in the natural fermentation group was considerably more complex. The total abundance of Cyanobacteria (49%), Proteobacteria (26%), Actinobacteria (7.9%), Chloromyces (4.4%), and Acidobacteria (2.8%) leads to >90% of the total abundance of all phyla. The results indicated that Firmicutes had become the dominant bacteria in the microbial fermentation system on the third day of fermentation in the added-bacteria fermentation group, whereas the natural fermentation group was still in the period of mixed strain fermentation; further, no dominant bacteria took the majority proportion. This indicates that the addition of Lacticaseibacillus paracasei HD1.7 can increase the abundance of Firmicutes and decrease the abundance of other bacteria (e.g., Cyanobacteria and Actinobacteria) in the system.

The species whose abundance ratio was <0.01% in all samples were divided into others. The composition changes of the bacteria community at the genus level between added bacteria and natural fermented pickled cucumber on the third day of fermentation are shown in Figure 2b. After adding Lacticaseibacillus paracasei HD1.7, the bacterial genera in the cucumber fermentation system had obvious succession. The top six genera in the natural fermentation group were the following: norank_f_norank_o_Chloroplast (49%), others (41%), Monas (2.9%), Pseudomonas (2.6%), norank_f_Mitochondria (2.5%), and Lysobacter (2.2%). The abundance of Lactobacillus alone in the added-bacteria fermentation group accounted for 97%, which further indicated that dominant bacteria genera appeared in the microbial system of pickled cucumber at this time. The free amino acids and fatty acids in vegetables can be degraded into volatile compounds with fermentation, and the amino acids and free fatty acids produced by proteolytic reactions are degraded through the mediation of chemical and microbial reactions during fermentation, adding unique flavor to fermented vegetables [16]. Pseudomonas will secrete tyrosinase in the fermentation process, and too much tyrosinase will produce melanin, which will cause adverse effects on the appearance of the product [17]. However, Pseudomonas will also secrete some other amino acids in the late fermentation, which will have a certain impact on the flavor of the product [18].

### 3.2. Network Characteristics of Pickled Cucumber

The collinear network map mainly reflects the co-existence relationship of species in environmental samples. As shown in Figure 2c, the network diameter is 3, and the shortest path length between each node in the network is 1.99 on average. The natural group has 22 horizontal categories, including 2 categories common to the added-bacteria group: Proteobacteria and Firmicutes.

The relationship between micro-organisms in naturally fermented pickled cucumber samples was studied using a single-factor correlation network diagram. The network map revealed significant correlations between different genera of bacterial communities in the sample. We performed a co-occurrence network unifactor analysis for the 50 most abundant genera in the pickled cucumber sample. As shown in Figure 2d, there are 50 nodes (genera) in the microbial network. There are eight groups of bacteria, including Acidobacteria, Firmicutes, Proteobacteria, Blastomonas, Chloroflexi, Deinococcota, Actinobacteria, and Cyanobacteria. Actinobacteria and Proteobacteria are widely distributed, accounting for 60% of the total number of segments. In the network diagram, the most connected nodes (genera) are unclassified_f_Rhizobiaceae, Phenylobacterium, and Microbacterium, which are all related to 16 nodes, and their clustering coefficient is 0.908333. These three genera are also strongly related to other genera. In addition, the genera that are relevant in the network diagram are Pseudomonas, Bacillus, and Lactobacillus. These genera all have a clustering coefficient of 1. The red line represents the positive correlation, the green line represents the negative correlation, the color of the connecting line represents the size of the correlation coefficient, and the color of the same node represents the same gate. In the figure, except for the negative correlation between Lactobacillus and three genera in the bacterial group in the sample, the other 182 sides are positively correlated, suggesting that the growth of Lactobacillus can reduce the abundance level of other bacterial genera. Lactobacillus is the dominant microbe in the fermentation process, which can use the glucose in cucumbers to produce rich lactic acid, which is fermented to produce carbon dioxide and ethanol, volatile compounds, and alcohols that can provide the distinctive taste and aroma of pickled cucumber products [19]. Relevant studies have shown that organic acids and bacteriocins produced by the metabolism of lactic acid bacteria can inhibit the growth of miscellaneous bacteria to some extent [20]. Bacillus is one of the most common genus of spoilage bacteria in pickled vegetables. When studying the classification of food spoilage bacteria, Andrses et al. [21] found that Bacillus is the main spoilage strain that causes a short shelf life of baking products and dairy products. These results indicate that the addition of Lacticaseibacillus paracasei HD1.7 can inhibit the emergence of bacteria represented by Bacillus in the cucumber fermentation system. Our laboratory has carried out a large number of systematic studies on the bacteriocins and population effects produced by Lacticaseibacillus paracasei HD1.7, and it has been proven that Lacticaseibacillus paracasei HD1.7 is a natural preservative with great application potential [22]. Other studies have shown that Bacillus in the co-culture system of Bacillus and Lacticaseibacillus paracasei HD 1.7 is beneficial to the production of bacteriocin in Lacticaseibacillus paracasei HD 1.7, and some Bacillus can be inhibited by bacteriocin in the co-culture system [23].

### 3.3. Discovery of Biomarkers in Pickled Cucumber

**Figure 3:**
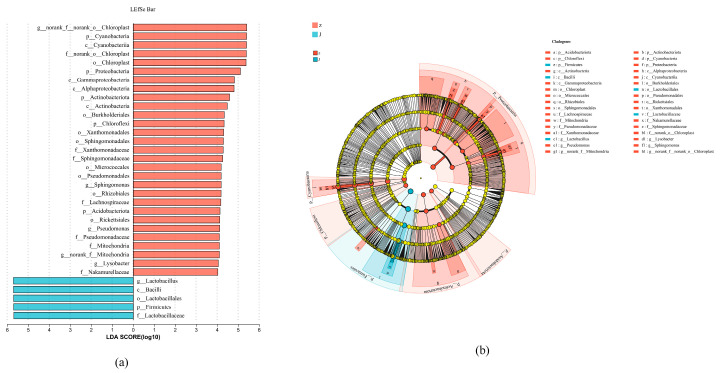
Analysis of biomarkers in pickled cucumber. (**a**) LDA analysis of J&Z samples (*p* < 0.05). (**b**) LEfSe multilevel species hierarchy tree analysis of J&Z samples (*p* < 0.05).

Linear discriminant analysis was used to analyze the differences in bacterial communities between different fermentation systems [24]. Through LDA analysis results (Figure 3a), it can be observed that the number of significantly different species in the fermentation system with Lacticaseibacillus paracasei HD1.7 bacteria is lower than natural fermentation, but the influence degree of different species is higher than natural fermentation.

From the LEfSe multilevel species hierarchy tree diagram (Figure 3b), biomarkers in pickled cucumber samples all appeared in the branch diagram, and micro-organisms with an LDA score > 4 were selected for labeling. From the perspective of dependency level, there are five kinds of natural fermentation biomarkers: norank_f_norank_o_Chloroplast, Lysobacter, Pseudomonas, norank_f_Mitochondria, and Sphingomonas. The microbial diversity of the added-bacteria fermentation was low, and only one biomarker was found, which was Lactobacillus. The biomarker Pseudomonas appeared in the natural group, and Pseudomonas has high proteolytic activity and high salt-tolerant reverse concentration tolerance, which can accelerate the fermentation rate of cucumber in the natural group. Some studies have shown that during the fermentation of cucumber, the hydrolyzed protein can provide nutrients needed by micro-organisms and improve fermentation efficiency [25].

### 3.4. Analysis of the Metabolic Pathway of Pickled Cucumber

**Figure 4:**
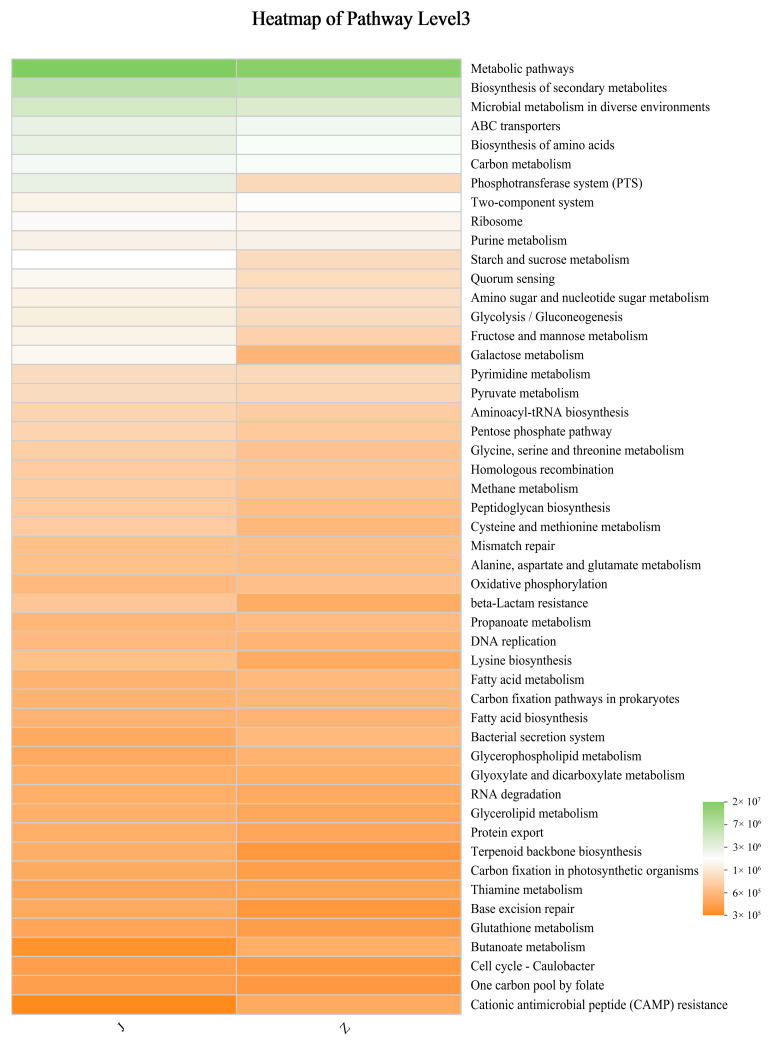
Microbial metabolic pathways of pickled cucumber.

There are six types of metabolic pathways in the fermentation process of pickled cucumber. The most abundant metabolic pathways in pickled cucumber were metabolism, environmental information processing, and genetic information processing. In particular, metabolic pathways, the biosynthesis of secondary metabolites, and microbial metabolism in diverse environments were generated. Heat maps were used to show the distribution of KEGG functional abundance in different samples, and the distribution of the main dominant functions in different samples was visually demonstrated. It was found that the metabolic pathway of pickled cucumber in group J was higher than that in group Z. In the fermentation process of pickled cucumber, alcohol compounds account for the largest proportion of volatile components, and an appropriate amount of high alcohol is an indispensable aroma and flavor substance in food, which plays a role in setting off the overall aroma, but too much high alcohol content will lead to a decline in food taste. 2-Propanol is mainly produced in butanoate metabolism. Both propane and acetone can produce 2-propanol under the catalysis of enzymes. The abundance of group J in butanoate metabolism is significantly lower than that of group Z. These results reveal that added-bacteria fermentation inhibits the production of 2-propanol in butanoate metabolism, thereby affecting metabolic pathways. Lactic acid bacteria play a leading role in the fermentation process of pickled cucumber and can break down some of the carbon sources and nitrogen sources to produce some nutrients for metabolic pathways [26]. The abundance of microbial diversity in pickled cucumber was closely related to group J (Figure 4).

### 3.5. Study on the GC–IMS Fingerprint of Pickled Cucumber

**Figure 5:**
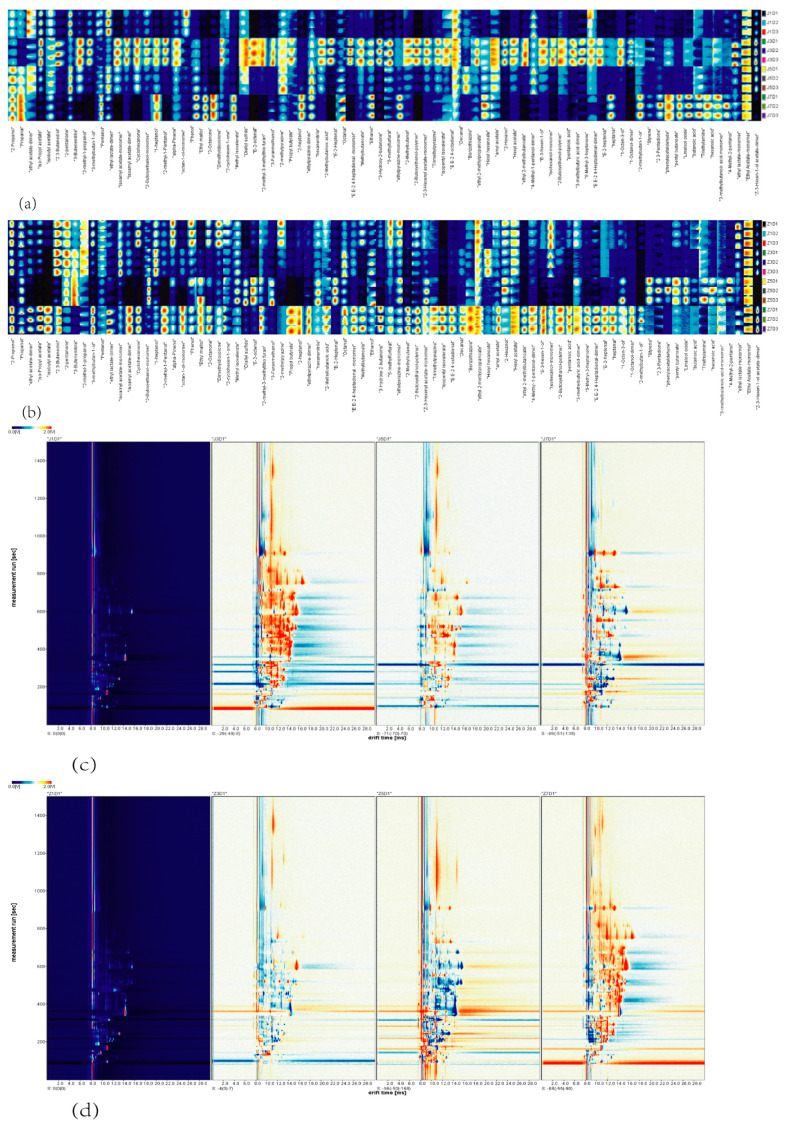
Analysis of volatile compounds. (**a**) Fingerprint of selected volatile organic compounds in the two-dimensional map of GC–IMS (added-bacteria fermentation group). (**b**) Fingerprint of selected volatile organic compounds in the two-dimensional map of GC–IMS (natural fermentation group). (**c**) Gas chromatographic–ion mobility spectrometry (GC–IMS) two-dimensional map subtraction map with the sample on the first day as reference (added-bacteria fermentation group). (**d**) GC–IMS two-dimensional map subtraction map with the sample on the first day as reference (natural fermentation group).

FavourSpec^®^GC–IMS (the Analysis Sensor System MBH (G.A.S.) instrument of the Dortmund Association, Germany, Purchased from Hanon Advanced Technology Group Co., Ltd.) was used to analyze the volatile organic compound fingerprints of pickled cucumber, and the VOC fingerprints are displayed in Figure 5a,b. Seventy-five volatile organic compounds were identified from naturally fermented pickled cucumber (as shown in Table 1), and sixty volatile organic compounds were identified from added-bacteria fermented pickled cucumber (as shown in Table 2). Ethyl acetate, isoamyl acetate, 2-butoxyethanol, ethylpyrazine, and 2,4-heptadienal compounds, due to the presence of both monomers and dimers, have double peaks in the fingerprint.

Of the volatile components detected, esters and alcohols are the most common groups. Through the preliminary analysis of fingerprints, it was found that the composition of volatile substances in pickled cucumber samples was basically the same, but with the change in fermentation time, there were obvious differences in the concentration of several volatile substances between natural fermentation and added-bacterial fermentation. In the current study, it can be seen that the content of esters is low in the early stage of cucumber fermentation and becomes a relatively high proportion of volatile substances in the middle and late stages of fermentation. Ethyl acetate, iso-propyl acetate, isobutyl acetate, and propyl butyrate in pickled cucumber increased significantly after fermentation with bacteria. Ethyl acetate has a natural fruit aroma. It is widely used in food processing. Related studies have shown that most esters have a unique odor; for example, butyl acetate, propyl butyrate, and ethyl 2-methylpropionate are considered to have a fruit aroma, and butyl butyrate and 3-methylbutyl acetate have a banana odor [27]. In the present study, the concentration of many alcohols in fermentation with bacteria is significantly reduced compared with that in the natural fermentation group, such as 2-propanol, 2,3-butanediol, and 2-methyl-1-propanol. The excessive presence of this polyol will significantly deteriorate the taste and aroma of pickled cucumber. It has a certain negative impact on pickled cucumber [28]. After adding Lacticaseibacillus paracasei HD1.7 to broth, the concentration of some alcohols was reduced, and the product quality of pickled cucumber was improved. In the current study, high aldehydes were detected in all eight groups of natural and added-bacteria fermentation samples, but the types and contents of aldehydes in the added-bacteria fermentation group were more abundant than those in the natural fermentation group. For example, 2,4-heptadienal and 2-methylbutana were significantly increased after fermentation with added bacteria. E-2-heptenal is a kind of odor compound. The content of E-2-heptenal in pickled cucumber decreased after fermentation with bacteria, suggesting that fermentation with bacteria can have a beneficial effect on the flavor of pickled cucumber. Chen et al. [29] found that during Douchi fermentation, the oxidation and decomposition of lipids can produce aldehydes. The aroma of aldehydes is mainly related to a sweet, fruity, nutty, and caramel taste, so aldehydes are considered to be relevant ingredients that can improve flavor quality. In the present study, it was found that after the addition of Lacticaseibacillus paracasei HD1.7, the types and contents of ketones and alcohols in pickled cucumber were further improved in the middle and late fermentation, which was consistent with the results of the above literature. Studies have shown that lactic acid bacteria fermentation can increase the content of physiological active substances in fruits and vegetables, such as phenols and flavonoids, and improve antioxidant activity [30]. Zhou et al. [31] fermented kiwi fruit with L. plantarum and found that the total phenolic compound content increased from 1.03 to 1.68 g/kg, and the total flavonoid content increased from 217.5 mg/kg to 425.5 mg/kg. Mandha et al. [32] found that the contents of alcohols, ketones, monoterpenoids, and furans in watermelon juice fermented by S. pentosus increased, whereas the contents of aldehydes and alkanes decreased. Fonseca et al. [33] found that both single lactic acid bacteria fermentation and co-culture fermentation of two kinds of lactic acid bacteria increased the content of alcohol and ketone compounds in passion fruit juice, which was conducive to maintaining the color stability of the juice during its shelf life. Sensory analysis showed that the fermented samples were mainly related to salty, sour, bitter, and sweet tastes. The acids in this study, such as butyric acid and isovaleric acid, mainly appeared on the seventh day of bacterial addition and natural fermentation, and these acids would give fermented food a bad flavor. Majority acids (except butyric acid) are found only at the end of the fermentation [34].

We can find that group J1 (samples on the first day of fermentation in the bacteriated group) and group Z1 (samples on the first day of fermentation in the natural group) themselves have fewer flavor substances, and more flavor substances are released after curing, such as isoamyl isovalerate, 2-methylbutyrate ethyl ester, propyl butyrate and isoamyl acetate, 5-methyl-3-heptanone, 2-methylbutyraldehyde, and 2,3,5-trimethylpyrazine. In groups J3 (the third day of fermentation with added bacteria) and Z7 (the seventh day of natural fermentation), a high content of characteristic volatile components (high concentration components) was found, among which ketones and phenols were more abundant than other samples, which could be inferred that their corresponding flavor and taste were more abundant, suggesting that the addition of lactic acid bacteria can effectively shorten the fermentation time, improve the fermentation efficiency, and contribute to the formation of the product flavor of pickled cucumber [35].

GC–IMS analysis of natural and cultured fermentation samples obtained two-dimensional graphs as shown in Figure 5c,d. As shown in the figure, in the two-dimensional graphs after subtraction treatment, only a few blue dots appeared in the samples on the third day of cultured fermentation (J3D1, the second picture on the left of Figure 5c), and most of the rest were red dots, suggesting that in terms of concentration of volatile substances, compared with the first day, the samples on the third day of fermentation had a greater advantage, and the concentration of most of the same volatile substances was higher than that on the first day. Blue spots gradually increased on the fifth and seventh days, but in the natural group, the area with more red spots appeared on the seventh day of fermentation (Z7D1, the fourth picture on the left of Figure 5d). This result indicated that added-bacteria fermentation could affect the production process of volatile compounds, and it was reasonable to predict that added-bacteria fermentation could shorten the fermentation cycle of cucumber and save time and cost.

### 3.6. Correlation Analysis of Bacterial Communities and Flavor-Volatile Components in Pickled Cucumber

**Figure 6:**
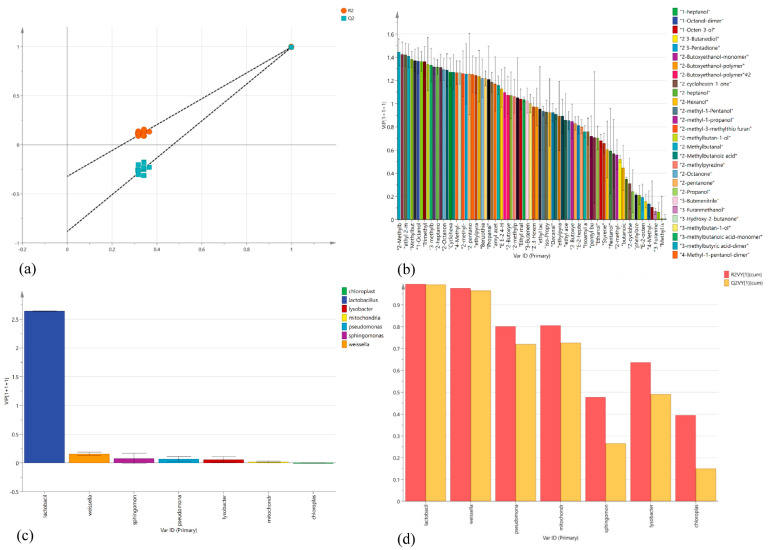
O2PLS model. (**a**) Permutations plot for O2PLS models. (**b**) Permutations plot for O2PLS models (microbial volatile organic components). (**c**) Permutations plot for O2PLS models (biomarker). (**d**) X/Y overview plot for O2PLS.

An O2PLS model was established using SIMCA-14.1 to forecast potential correlations between core bacterial communities and flavor-volatile components. The missing value tolerance for variables and observations is set to the default 50%. By using the bacterial community abundance values shown by high-throughput sequencing and volatile metabolite-related data obtained by GC–IMS, the predicted VIP effect of R2X = 0.991, R2Y = 0.994, and Q2 = 0.991 was obtained with similarity to the original model as the *X*-axis and R2 and Q2 values as the *Y*-axis (Figure 6a). All three values were close to 1. The results reveal that the model has a good prediction effect.

Figure 6b shows the VIP diagram of O2PLS model fitting, where the horizontal co-ordinate is volatile metabolites and the vertical co-ordinate is variable importance in projection, which is used to describe the importance of volatile metabolites to model fitting. Variables with a larger VIP are more relevant. The results indicated that 42 volatile metabolites were significantly correlated with model fitting (VIP ≥ 1). According to the importance of bacteria to flavor components, the VIP vector of the core flora was further verified (Figure 6b,c), and it was found that Lactobacillus was the most abundant bacterial group, indicating that it was the most important contributor to the fitting generation of the O2PLS model among biomarkers.

Figure 6d shows an X–Y summary of the fit based on the O2PLS model, where the horizontal co-ordinate is the bacterial community’s name and the vertical co-ordinate is the cumulative R2 and Q2 values for each variable in O2PLS. Good variable conditions for modeling are red bars (R2) and yellow bars (Q2) ≥ 0.5, where R2 represents the ability to explain variable changes using prediction components, whereas Q2 represents the ability for variables to be predicted, and Q2 is estimated by cross-validation. The results revealed that all bacterial communities except Sphingomonas and norank_f_norank_o_Chloroplast were good variables for the model.

**Figure 7:**
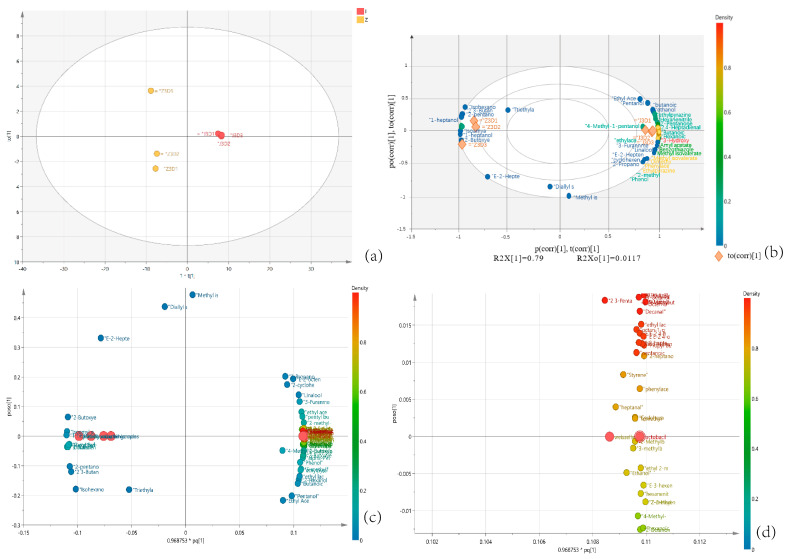
Correlations between bacterial taxa and metabolites. (**a**) Scores scatter plot t1 vs. t1o plot O2PLS. (**b**) Biplot loadings sample and microbial volatile organic component of O2PLS. (**c**) X (microbial volatile organic components) vs. Y (biomarker) plot O2PLS. (**d**) Biplot loadings p and scores t of O2PLS (expanded edition).

Figure 7a shows the scatter distribution between the samples. Observing the *X*-axis (t1 axis), it can be found that the groups in this study have separation ability, which indicates that the volatile metabolites and biomarkers in the natural fermentation and added-bacteria fermentation groups are significantly different. BioPlot can show the relationship between load and score simultaneously. A correlation matrix was used to analyze the correlation coefficient between core bacteria and volatile components, and Pearson correlation analysis was used to verify the calculated results (Figure 7b). Ten volatile metabolites were found to be significantly related to the natural group samples, whereas 60 volatile metabolites were found to be closely related to the bacterial group samples. Among them, the representative volatile components with high abundance in the natural group include three alcohols, one nitrile, one aldehyde, one ester, and one ketone. The representative volatile components with a high abundance of Lactobacillus group are six esters, two aldehydes, and one ketone, which proves that the exogenous addition of Lactobacillus can increase the variety and abundance of volatile metabolites in the sample.

Figure 7c predicts the relationship between X (volatile metabolites) and Y (biomarkers), and it is found that volatile metabolites in the added-bacteria group have a more complex association with bacterial community correlation than in the natural group. The X and Y scatter maps were enlarged (Figure 7d), in which Weissella and Lactobacillus in the added-bacteria group were closely positively correlated with nine alcohols, six esters, five aldehydes, four acids, three ketones, and one pyrazine. Pseudomonas and norank_f_Mitochondria in the natural group showed a close positive correlation with four kinds of alcohols, two kinds of esters, one kind of aldehyde, and one kind of nitrile. It can be seen that Weissella and Lactobacillus in the Lactobacillus group are correlated with the most volatile metabolites, and the diverse aroma of pickled cucumber is due to the diversity of their core bacterial species. The lactic acid flora can promote the degradation of amino acids, which can enrich the aroma composition of pickled cucumber [36].

Microbial metabolism is the main reason to promote fermentation, which is the formation of some typical fermented flavors that are closely related [37]. Lactobacillus is the most significant contributor to the production of volatile substances during cucumber fermentation (VIP > 1.0), which is in line with the research results of Rao and Liang et al. Rao et al. [38] showed that Lactobacillus is positively correlated with the production of aromatic ester in fermented mustard products and pickled radishes. Liang et al. [39] showed that the co-inoculation of two strains could increase the contents of alcohol, ester, and nitrile in sauerkraut. The results of this study reveal that Lactobacillus can change the bacterial community structure in the fermentation system while maintaining the quality of pickled cucumber and displaying its special aroma characteristics. Long-term storage will reduce the microbial activity in vegetables, and will reduce the number of lactic acid bacteria, but the effect is not significant [40]. Cucumbers after fermentation, compared with unfermented cucumbers, not only increase the shelf life, but also increase a lot of probiotics; lactic acid bacteria are one of the main members, they have a certain role in regulating the human intestinal flora [41]. Nitrite, which is toxic and carcinogenic, is produced by nitrate-reducing bacteria during food fermentation, especially in the early stages of fermentation. The content of nitrite in Lacticaseibacillus paracasei HD1.7 fermented samples was significantly lower than that in the control, indicating that Lacticaseibacillus paracasei HD1.7 may have inhibitory activity against nitrate-reducing bacteria [42]. The inoculation of Lacticaseibacillus paracasei HD1.7 during cucumber fermentation can reduce the content of nitrite and provide a guarantee for product safety. Numerous studies have shown that both Weissella and Lactobacillus have beneficial effects on vegetable fermentation; for example, pickle fermentation is dominated by Leuconostoc, Weissella, and Lactobacillus [43]. Weissella plays an important role in pickle fermentation, as does Lactobacillus [44]. In addition, it has been found that the exopolymer secreted by Weissella can enhance the outstanding viscosity and polymerization properties of polymers in fermented foods, and has the potential to improve the stability and sensory properties of products as a natural thickener and emulsifying stabilizer [45]. Luo et al. [46] used a mixture of Leuconostoc, Weissella, and Lactobacillus to ferments pickles, and the results showed that it can minimize nitrite content. Therefore, the addition of Lacticaseibacillus paracasei HD1.7 can not only increase the proportion of Lactobacillus in the fermentation system, but also contribute to the increase of the proportion of the Weissella system, helping pickles to produce better product quality and flavor characteristics. In addition to Lactobacillus, there are other microbial growth metabolites that help ferment vegetables to produce their distinctive aroma. Studies have shown that *E. coli* in fermented vegetable systems can decompose glucose into lactic acid, acetic acid, and carbon dioxide, while Propionibacterium has the ability to convert carbohydrates into acetic acid, propionic acid, and carbon dioxide [47].

## 4. Conclusions

This study investigated the microbial diversity and volatile substances of pickled cucumber during pickling. Seventy-five volatile organic compounds were identified from naturally fermented pickled cucumber, and sixty volatile organic compounds were identified from added-bacteria-fermented pickled cucumber. In the early stage of pickled cucumber fermentation, the content of esters is low, and in the middle and late stage of fermentation, esters become relatively volatile substances. The high-throughput sequencing results indicated that the addition of *Lacticaseibacillus paracasei* HD1.7 could reduce the bacterial community diversity in the pickled cucumber. Compared with natural fermentation, added-bacterial fermentation can increase the proportion of *Lactobacillus* and effectively reduce the content of miscellaneous bacteria. The O2PLS model showed that volatile metabolites in the added-bacteria group had more complex associations with bacterial community correlation than those in the natural group. This study laid a theoretical foundation for improving the quality of pickled cucumber.

## Figures and Tables

**Table 1:** Volatile flavor compounds identified in naturally fermented cucumber.

Count	Compound	CAS#	Formula	MW	RI	Rt (s)	Dt (RIPrel)
1	2-Propanol	C67630	C_3_H_8_O	60.1	487.5	145.56	1.18
2	Propanal	C123386	C_3_H_6_O	58.1	473.9	140.03	1.15
3	Ethyl acetate	C141786	C_4_H_8_O_2_	88.1	616.5	198.16	1.34
4	Iso-propyl acetate	C108214	C_5_H_10_O_2_	102.1	651.3	214.12	1.47
5	Isobutyl acetate	C110190	C_6_H_12_O_2_	116.2	755.2	291.58	1.62
6	2-Methyl-1-propanol	C78831	C_4_H_10_O	74.1	657.6	217.35	1.17
7	2,3-Butanediol	C513859	C_4_H_10_O_2_	90.1	780.3	316.76	1.37
8	3-Methylbutan-1-ol	C123513	C_5_H_12_O	88.1	756.4	292.77	1.24
9	Pentanol	C71410	C_5_H_12_O	88.1	781	317.55	1.25
10	Ethyl lactate	C97643	C_5_H_10_O_3_	118.1	801.9	339.23	1.54
11	1-Pentanol, 2-methyl-	C105306	C_6_H_14_O	102.2	828.2	368.20	1.30
12	Isoamyl acetate	C123922	C_7_H_14_O_2_	130.2	871.7	422.91	1.31
13	Isoamyl acetate	C123922	C_7_H_14_O_2_	130.2	868.8	418.89	1.75
14	Cyclohexanone	C108941	C_6_H_10_O	98.1	893.7	455.48	1.15
15	3-Furanmethanol	C4412913	C_5_H_6_O_2_	98.1	979.5	609.64	1.11
16	1-Heptanol	C111706	C_7_H_16_O	116.2	975	601.47	1.39
17	2,4-Heptadienal	C4313035	C_7_H_10_O	110.2	985.9	621.30	1.61
18	α-pinene	C80568	C_10_H_16_	136.2	986.6	622.68	1.21
19	Octan-1-ol	C111875	C_8_H_18_O	130.2	1071.7	753.76	1.46
20	Phenol	C108952	C_6_H_6_O	94.1	987	623.27	1.08
21	Ethyl maltol	C4940118	C_7_H_8_O_3_	140.1	1187.5	909.37	1.20
22	2-Octanone	C111137	C_8_H_16_O	128.2	986.1	621.77	1.33
23	Dimethyldioxolane	C37830903	C_5_H_6_O_3_	114.1	948.2	551.47	1.17
24	2-Cyclohexen-1-one	C930687	C_6_H_8_O	96.1	914	489.20	1.11
25	Methyl isovalerate	C556241	C_6_H_12_O_2_	116.2	795.4	332.36	1.20
26	2-Octenal (E)	C2548870	C_8_H_14_O	126.2	1054.5	729.88	1.33
27	2-Methyl-3-(Methylthio)furan	C63012975	C_6_H_8_OS	128.2	957.5	568.78	1.11
28	2-Methylpyrazine	C109080	C_5_H_6_N_2_	94.1	845.5	388.65	1.40
29	Propyl butyrate	C105668	C_7_H_14_O2	130.2	922.2	503.68	1.69
30	2-Heptanol	C543497	C_7_H_16_O	116.2	921.7	502.79	1.72
31	Ethylpyrazine	C13925003	C_6_H_8_N_2_	108.1	920.2	500.11	1.51
32	Hexanenitrile	C628739	C_6_H_1_1N	97.2	879.7	434.35	1.26
33	2-Methylbutanoic acid	C116530	C_5_H_10_O_2_	102.1	883.3	439.57	1.20
34	Octanal	C124130	C_8_H_16_O	128.2	997.6	641.85	1.39
35	Methylbutanoate	C623427	C_5_H_10_O_2_	102.1	721.4	260.66	1.44
36	Ethanol	C64175	C_2_H_6_O	46.1	455.1	132.40	1.14
37	3-Hydroxy-2-butanone	C513860	C_4_H_8_O_2_	88.1	721.6	260.84	1.33
38	2-Butoxyethanol	C111762	C_6_H_14_O_2_	118.2	904.3	475.68	1.24
39	5-Methylfurfural	C620020	C_6_H_6_O_2_	110.1	959.1	571.87	1.13
40	Ethylpyrazine	C13925003	C_6_H_8_N_2_	108.1	916.6	493.82	1.13
41	2-Methylbutanal	C96173	C_5_H_10_O	86.1	680.7	230.54	1.40
42	(Z)-3-hexenyl acetate	C3681718	C_8_H_14_O_2_	142.2	997.7	643.07	1.31
43	Trimethylpyrazine	C14667551	C_7_H_10_N_2_	122.2	998.2	642.99	1.17
44	Isopentyl isovalerate	C659701	C_10_H_20_O_2_	172.3	1071.3	753.26	2.02
45	Decanal	C112312	C_10_H_20_O	156.3	1199.6	925.59	1.53
46	Benzothiazole	C95169	C_7_H_5_NS	135.2	1247.1	989.45	1.16
47	Ethyl 2-methylpropanoate	C97621	C_6_H_12_O_2_	116.2	751.8	288.35	1.20
48	Hexyl hexanoate	C6378650	C_12_H_24_O_2_	200.3	1378.3	1165.62	1.60
49	Amyl acetate	C628637	C_7_H_14_O_2_	130.2	912.9	487.34	1.33
50	2-Butoxyethanol	C111762	C_6_H_14_O_2_	118.2	904.3	472.70	1.65
51	2-Hexanol	C626937	C_6_H_14_O	102.2	795	331.96	1.29
52	Hexyl acetate	C142927	C_8_H_16_O_2_	144.2	997.6	641.97	1.42
53	Ethyl 2-methylbutanoate	C7452791	C_7_H_14_O_2_	130.2	856.9	403.05	1.66
54	4-Methyl-1-pentanol	C626891	C_6_H_14_O	102.2	849.6	393.81	1.62
55	1-Octanol	C111875	C_8_H_18_O	130.2	1073.3	755.97	1.89
56	(E)-3-hexen-1-ol	C928972	C_6_H_12_O	100.2	852.2	397.09	1.53
57	Isohexanol	C626891	C_6_H_14_O	102.2	850.6	395.03	1.31
58	Pentanoic acid	C109524	C_5_H_10_O_2_	102.1	893.4	454.96	1.50
59	3-Methylbutyric acid	C503742	C_5_H_10_O_2_	102.1	878.5	432.55	1.48
60	5-Methyl-3-heptanone	C541855	C_8_H_16_O	128.2	952.3	559.15	1.70
61	Heptanal	C111717	C_7_H_14_O	114.2	940.7	537.44	1.33
62	1-Octen-3-ol	C3391864	C_8_H_16_O	128.2	949.7	554.24	1.60
63	2-Methylbutan-1-ol	C137326	C_5_H_12_O	88.1	763.7	299.98	1.47
64	2,3-Pentadione	C600146	C_5_H_8_O_2_	100.1	662	219.70	1.30
65	Pentyl butanoate	C540181	C_9_H_18_O_2_	158.2	1056.2	732.36	1.41
66	Linalool oxide	C60047178	C_10_H_18_O_2_	170.3	1087	774.53	1.25
67	Butanoic acid	C107926	C_4_H_8_O_2_	88.1	853.9	399.26	1.38
68	2,4-Heptadienal	C4313035	C_7_H_10_O	110.2	976.5	604.15	1.19
69	Triethylamine	C121448	C_6_H_15_N	101.2	659.9	218.54	1.22
70	Hexanoic acid	C142621	C_6_H_12_O_2_	116.2	1016.4	673.06	1.64
71	3-Methylbutanoic acid	C503742	C_5_H_10_O_2_	102.1	854.5	400.02	1.22
72	4-Methyl-2-pentanol	C108112	C_6_H_14_O	102.2	767.8	304.14	1.28
73	Ethyl lactate	C97643	C_5_H_10_O_3_	118.1	801	338.29	1.14
74	Ethyl acetate	C141786	C_4_H_8_O_2_	88.1	596.4	189.86	1.10
75	3-Hexen-1-ol, acetate, (Z)	C3681718	C_8_H_14_O_2_	142.2	1017.5	674.80	1.81

**Table 2:** Volatile flavor compounds identified in added-bacteria fermentation cucumber.

Count	Compound	CAS#	Formula	MW	RI	Rt(s)	Dt (RIPrel)
1	Propanal	C123386	C_3_H_6_O	58.1	473.9	140.03	1.15
2	Iso-propyl acetate	C108214	C_5_H_10_O_2_	102.1	651.3	214.12	1.47
3	2,3-Butanediol	C513859	C_4_H_10_O_2_	90.1	780.3	316.76	1.37
4	2-Methyl-1-propanol	C78831	C_4_H_10_O	74.1	657.6	217.35	1.17
5	2-Pentanone	C107879	C_5_H_10_O	86.1	678.6	229.23	1.13
6	3-Butenenitrile	C109751	C_4_H_5_N	67.1	642.5	209.83	1.13
7	Pentanol	C71410	C_5_H_12_O	88.1	781	317.55	1.25
8	Isoamyl acetate	C123922	C_7_H_14_O_2_	130.2	871.7	422.91	1.31
9	Isoamyl acetate	C123922	C_7_H_14_O_2_	130.2	868.8	418.89	1.75
10	2-Butoxyethanol	C111762	C_6_H_14_O_2_	118.2	906.1	475.68	1.24
11	1-Heptanol	C111706	C_7_H_16_O	116.2	975	601.47	1.39
12	2,4-Heptadienal	C4313035	C_7_H_10_O	110.2	985.9	621.30	1.61
13	α-pinene	C80568	C_10_H_16_	136.2	986.6	622.68	1.21
14	Phenol	C108952	C_6_H_6_O	94.1	987	623.27	1.08
15	Ethyl maltol	C4940118	C_7_H_8_O_3_	140.1	1187.5	909.37	1.20
16	Dimethyldioxolone	C37830903	C_5_H_6_O_3_	114.1	948.2	551.47	1.17
17	2-Cyclohexen-1-one	C930687	C_6_H_8_O	96.1	914	489.20	1.11
18	Methyl isovalerate	C556241	C_6_H_12_O_2_	116.2	795.4	332.36	1.20
19	Diallyl sulfide	C592881	C_6_H_10_S	114.2	840.5	382.60	1.11
20	2-Octenal (E)	C2548870	C_8_H_14_O	126.2	1054.5	729.88	1.33
21	2-Methyl-3-(Methylthio)furan	C63012975	C_6_H_8_OS	128.2	957.5	568.78	1.11
22	3-Furanmethanol	C4412913	C_5_H_6_O_2_	98.1	979.5	609.64	1.11
23	2-Methylpyrazine	C109080	C_5_H_6_N_2_	94.1	845.5	388.65	1.40
24	Ethylpyrazine	C13925003	C_6_H_8_N_2_	108.1	920.2	500.11	1.51
25	Hexanenitrile	C628739	C_6_H_1_1N	97.2	879.7	434.35	1.26
26	2-Methylbutanoic acid	C116530	C_5_H_10_O_2_	102.1	883.3	439.57	1.20
27	(E)-2-Heptenal	C18829555	C_7_H_12_O	112.2	949.7	554.27	1.25
28	Octanal	C124130	C_8_H_16_O	128.2	997.6	641.85	1.39
29	Ethanol	C64175	C_2_H_6_O	46.1	455.1	132.40	1.14
30	5-Methylfurfural	C620020	C_6_H_6_O_2_	110.1	959.1	571.87	1.13
31	Ethylpyrazine	C13925003	C_6_H_8_N_2_	108.1	916.6	493.82	1.13
32	2-Butoxyethanol	C111762	C_6_H_14_O_2_	118.2	906.4	476.30	1.59
33	(Z)-3-hexenyl acetate	C3681718	C_8_H_14_O_2_	142.2	997.7	643.07	1.31
34	Trimethylpyrazine	C14667551	C_7_H_10_N_2_	122.2	998.2	642.99	1.17
35	(E, E)-2,4-octadienal	C30361285	C_8_H_12_O	124.2	1118.6	816.93	1.27
36	Decanal	C112312	C_10_H_20_O	156.3	1199.6	925.59	1.53
37	Benzothiazole	C95169	C_7_H_5_NS	135.2	1247.1	989.45	1.16
38	Ethyl 2-methylpropanoate	C97621	C_6_H_12_O_2_	116.2	751.8	288.35	1.20
39	Hexyl hexanoate	C6378650	C_12_H_24_O_2_	200.3	1378.3	1165.62	1.60
40	Amyl acetate	C628637	C_7_H_14_O_2_	130.2	912.9	487.34	1.33
41	2-Hexanol	C626937	C_6_H_14_O	102.2	795	331.96	1.29
42	4-Methyl-1-pentanol	C626891	C_6_H_14_O	102.2	849.6	393.81	1.62
43	2-butoxyethanol	C111762	C_6_H_14_O_2_	118.2	904.3	472.70	1.65
44	5-methyl-3-heptanone	C541855	C_8_H_16_O	128.2	952.3	559.15	1.70
45	Heptanal	C111717	C_7_H_14_O	114.2	940.7	537.44	1.33
46	1-Octen-3-ol	C3391864	C_8_H_16_O	128.2	949.7	554.24	1.60
47	Styrene	C100425	C_8_H_8_	104.2	881.1	436.21	1.44
48	2,3-Pentadione	C600146	C_5_H_8_O_2_	100.1	662	219.70	1.30
49	Phenylacetaldehyde	C122781	C_8_H_8_O	120.2	1018	675.58	1.25
50	Pentyl butanoate	C540181	C_9_H_18_O_2_	158.2	1056.2	732.36	1.41
51	Linalool oxide	C60047178	C_10_H_18_O_2_	170.3	1087	774.53	1.25
52	Butanoic acid	C107926	C_4_H_8_O_2_	88.1	853.9	399.26	1.38
53	2,4-Heptadienal	C4313035	C_7_H_10_O	110.2	976.5	604.15	1.19
54	Triethylamine	C121448	C_6_H_15_N	101.2	659.9	218.54	1.22
55	Hexanoic acid	C142621	C_6_H_12_O_2_	116.2	1016.4	673.06	1.64
56	3-Methylbutanoic acid	C503742	C_5_H_10_O_2_	102.1	854.5	400.02	1.22
57	4-Methyl-2-pentanol	C108112	C_6_H_14_O	102.2	767.8	304.14	1.28
58	Ethyl lactate	C97643	C_5_H_10_O_3_	118.1	801	338.29	1.14
59	Ethyl acetate	C141786	C_4_H_8_O_2_	88.1	596.4	189.86	1.10
60	3-Hexen-1-ol, acetate, (Z)	C3681718	C_8_H_14_O_2_	142.2	1017.5	674.80	1.81

## Data Availability

The original contributions presented in the study are included in the article, further inquiries can be directed to the corresponding authors.
